# Incidence, Disease Severity, and Follow-Up of Influenza A/A, A/B, and B/B Virus Dual Infections in Children: A Hospital-Based Digital Surveillance Program

**DOI:** 10.3390/v14030603

**Published:** 2022-03-14

**Authors:** Patrick E. Obermeier, Lea D. Seeber, Maren Alchikh, Brunhilde Schweiger, Barbara A. Rath

**Affiliations:** 1 Vienna Vaccine Safety Initiative, Infectious Diseases & Vaccines, D-10437 Berlin, Germany; p.e.obermeier@gmail.com (P.E.O.); lea.seeber@gmail.com (L.D.S.); maren.alchikh@hotmail.de (M.A.); 2 Laboratoire Chrono-Environnement LCE, UMR CNRS 6249, Université Bourgogne Franche-Comté, F-25000 Besançon, France; 3 National Reference Center for Influenza, Robert Koch-Institute, D-13353 Berlin, Germany; bruni.schweiger@gmx.de

**Keywords:** coinfection, influenza A virus, influenza B virus, lineage, subtype, vaccine-preventable disease, disease severity, point-of-care testing, bedside testing, mobile health

## Abstract

Influenza virus (IV) coinfection, i.e., simultaneous infection with IV and other viruses, is a common occurrence in humans. However, little is known about the incidence and clinical impact of coinfection with two different IV subtypes or lineages (“dual infections”). We report the incidence, standardized disease severity, and follow-up of IV dual infections from a hospital-based digital surveillance cohort, comprising 6073 pediatric patients fulfilling pre-defined criteria of influenza-like illness in Berlin, Germany. All patients were tested for IV A/B by PCR, including subtypes/lineages. We assessed all patients at the bedside using the mobile ViVI ScoreApp, providing a validated disease severity score in real-time. IV-positive patients underwent follow-up assessments until resolution of symptoms. Overall, IV dual infections were rare (4/6073 cases; 0.07%, incidence 12/100,000 per year) but showed unusual and/or prolonged clinical presentations with slightly above-average disease severity. We observed viral rebound, serial infection, and B/Yamagata-B/Victoria dual infection. Digital tools, used for instant clinical assessments at the bedside, combined with baseline/follow-up virologic investigation, help identify coinfections in cases of prolonged and/or complicated course of illness. Infection with one IV does not necessarily prevent consecutive or simultaneous (co-/dual) infection, highlighting the importance of multivalent influenza vaccination and enhanced digital clinical and virological surveillance.

## 1. Introduction

Influenza virus (IV) infection poses a serious health threat, especially to particular risk groups, including immunocompromised, elderly, and young individuals [1,2,3].

According to the latest International Committee on Taxonomy of Viruses’ (ICTV) Virus Taxonomy Release ratified in March 2021, the *Orthomyxoviridae* family comprises seven genera and nine species, including Influenza A virus (IAV), Influenza B virus (IBV), Influenza C virus (ICV), and Influenza D virus (IDV) [4]. IDV and ICV may be associated with mild or asymptomatic infection in humans [5,6,7] whereas IAV and IBV infection can cause severe and fatal diseases [2,8]. IAVs are further divided into subtypes based on their surface proteins hemagglutinin (H) and neuraminidase (N), of which A(H1N1)pdm09 and A(H3N2) are currently circulating amongst humans. IBVs are further classified into two distinct lineages, B/Victoria and B/Yamagata [8]. IAV subtypes and IBV lineages are antigenically different.

While IV infection alone can lead to considerable disease burden, coinfection (s) may also contribute to disease severity [9]. Bacterial coinfections are likely to receive more attention as we can treat them with antibiotics [9,10,11]. In the absence of many licensed antivirals for clinical use, viral coinfection may be neglected. However, notably, viral coinfection in addition to influenza occurs frequently, especially in children [12,13]. However, our knowledge on the particular role of IV dual infections is sparse and mainly based on singular case reports instead of systematic investigation [14,15,16,17,18,19,20,21].

For the reliable investigation of disease severity associated with coinfections, including IV dual infections, the real-time application of uniform case criteria as well as a standardized and meta-analyzable disease severity measure provide immense advantages over the use of International Classification of Diseases (ICD) codes or retroactive case classification and traditional surrogates of disease severity, such as hospitalization or admission to the intensive care unit (ICU) [12,22,23]. To facilitate harmonization of infectious disease diagnoses in both research and clinical settings, the Vienna Vaccine Safety Initiative (ViVI, https://www.vi-vi.org, accessed on 3 March 2022), an international non-profit research organization, has developed a mobile health application for the standardized assessment of real-world patient data at the bedside, including a validated and meta-analyzable composite measure for disease severity scoring: the ViVI ScoreApp. This mobile health tool was user-tested in a previously published digital surveillance cohort for children presenting with pre-defined signs and symptoms of influenza-like illness (ILI) and respiratory infection in Berlin, Germany [11,12,23,24,25,26,27,28].

Within the framework of this inception cohort, we leveraged uniform case classification of ILI and respiratory infection, standardized longitudinal patient assessments, and a comparable disease severity measure in addition to comprehensive IV PCR testing to investigate

- The incidence of IV dual infections in children based on a hospital-based digital surveillance program with a known denominator of all ILI cases and
- Disease severity, symptoms, and detailed course of illness in children with IV dual infections

## 2. Materials and Methods

The investigation was conducted in the framework of a perennial prospective quality improvement (QI) and digital surveillance program at the Charité University Medical Center in Berlin, Germany, in collaboration with the National Reference Center for Influenza at the Robert Koch-Institute in Berlin between December 2009 and April 2015. The cohort comprised a total of 6073 patients (56% male, mean age: 2.6 years, median age 1.6 years, range 0–18.8 years) [23].

### 2.1. Institutional Review Board Statement

Ethical approval was granted by the Charité Institutional Review Board (IRB, EA 24/008/10). All measures were performed in accordance with the Declaration of Helsinki. Informed consent procedures were waived to enhance infection control and quality of care, given the non-interventional nature of the program. However, caregivers provided individual consent for all underage patients presented in detail in this report.

### 2.2. Patient Cohort

We screened all pediatric patients aged 0–18 years presenting to the emergency room or ward of the Charité University Medical Center for pre-defined criteria of influenza-like illness (ILI) and respiratory infection, i.e., fever and ≥1 respiratory symptom following a simplified ILI case definition endorsed by the World Health Organization (WHO) or a documented clinician diagnosis of ILI [11,12,23,24,25,28]. Upon entry into the cohort, a full body examination and structured interview regarding the current and previous patient history were performed by specifically trained QI staff, independently from routine care. At the same time, we obtained nasopharyngeal swabs from all patients for real-time PCR testing of IAV and IBV in addition to seven other major respiratory viruses as published previously [11,12,23,24,28]. On all patients testing positive for IAV and/or IBV, we followed up every other day until viral clearance and/or the resolution of symptoms. As part of quality improvement and enhanced infection control, we shared virological test results immediately with the respective clinical teams recommending swift isolation measures.

### 2.3. Assessment of Symptoms and Disease Severity

Clinical signs and symptoms of ILI and respiratory infection were assessed at the bedside, using the ViVI ScoreApp for real-time computation of the previously validated ViVI Disease Severity Score [12]. The score combines nine items reflecting uncomplicated disease (e.g., rhinitis) with 13 items reflecting complicated disease (e.g., prolonged fever) referring to WHO criteria [29]. Output values range from 0-48, with increasing values reflecting increasing disease severity. Data formats and terminologies are in full compliance with the Clinical Data Interchange Standards Consortium (CDISC) and regulatory requirements [11,12,30]. The ViVI ScoreApp is available for iOS and Android online: https://score.vi-vi.org, accessed on 3 March 2022.

For all patients with IV dual infections, we report baseline and maximum ViVI Scores as well as corresponding z-scores for comparison with previously published ViVI Scores of the entire cohort [12].

### 2.4. Virology Testing

We referred all nasopharyngeal swabs to the National Reference Center for Influenza at the Robert Koch Institute in Copan universal transport medium (UTM), usually on the day of collection [11,12,24,25,27]. Nasopharyngeal swabs were adjusted to 3 mL with sterile minimal essential medium (MEM) with HEPES (Gibco, BRL, Eggenstein, Germany) and 100 U/mL penicillin-streptomycin. RNA was extracted from 300 µL of the patient specimen using the MagAttract Viral RNA M48 Kit (Qiagen, Hilden, Germany) and eluted in 80 µL elution buffer. Alternatively, RNA was extracted using the MagNA Pure 96 DNA and Viral NA Small Volume Kit (Roche Deutschland Holding GmbH, Mannheim, Germany) from a 200 µL specimen with an elution volume of 50 µL. A volume of 25 µL of extracted RNA was subjected to cDNA synthesis applying 200 U M-MLV Reverse Transcriptase (Invitrogen, Karlsruhe, Germany) in a total volume of 40 µL. The cDNA samples were analyzed by real-time PCR for the presence of IAV and/or IBV, including subtype (A(H1N1) pdm09, H3N2) and lineage (Victoria, Yamagata) determination [31,32]. Reactions were performed using the Light Cycler 480 real-time PCR system (Roche Deutschland Holding GmbH, Mannheim, Germany).

Ct values were used to approximate viral load in this analysis [24]. Ct values obtained with IV-negative samples were defined as 45. The lower limit of detection for IV real-time PCR was determined to be 2–15 genome equivalents per reaction (95% detection probability), depending on the target gene [31].

### 2.5. Statistics

Descriptive statistics were performed using SPSS software. (version 22.0; IBM Corp., Armonk, NY, USA).

## 3. Results

**Figure 1:**
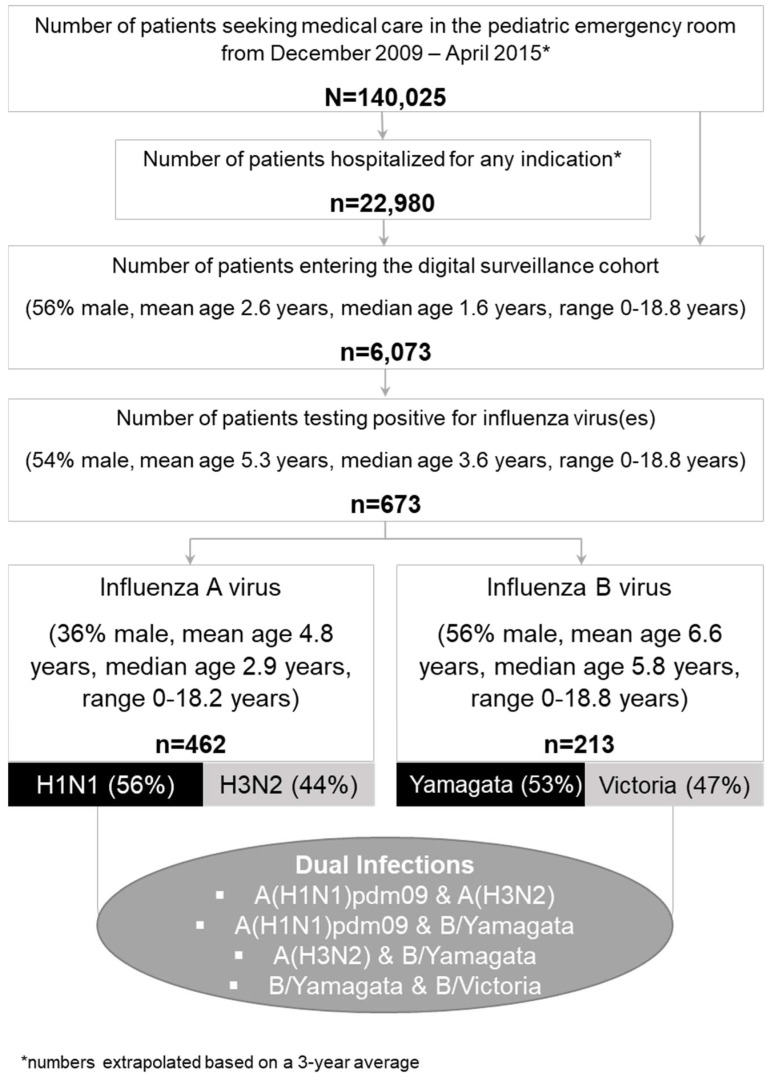
Flow chart, including patient and subtype/lineage details.

Of all 6073 patients fulfilling cohort entry criteria, a total of 673 (10%) tested positive for IV at baseline. Of those, 462 (69%) tested positive for IAV and 213 (31%) for IBV. Figure 1 provides a flow chart including further patient and subtype/lineage details. 

**Figure 2:**
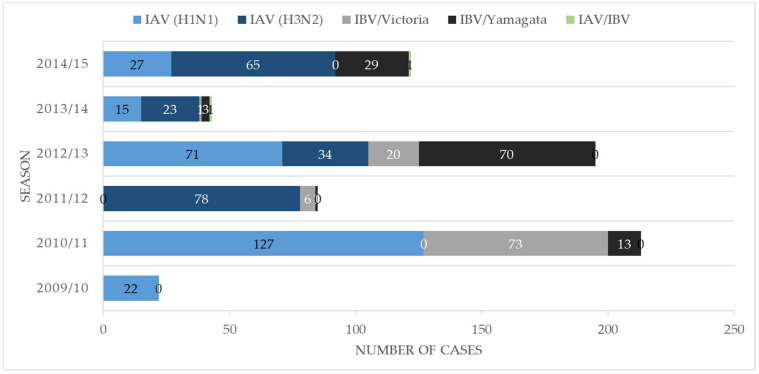
Numbers of IAV/IBV-positive cases, including subtypes and lineages per season.

We detected the largest proportion of IV-positive cases during the 2010/11 season between October and April. Yet, IBV was almost absent that year. Figure 2 depicts numbers of IAV/IBV-positive cases, including subtypes and lineages per season.

Overall, four patients out of 6073 (0.07%) presented with IV A/A, A/B, or B/B dual infections. This corresponds to an incidence rate of 12/100,000 ILI patients (*per annum*, as observed in an observation period of 65 months total).

### 3.1. Overview of Patient Characteristics

**Figure 3:**
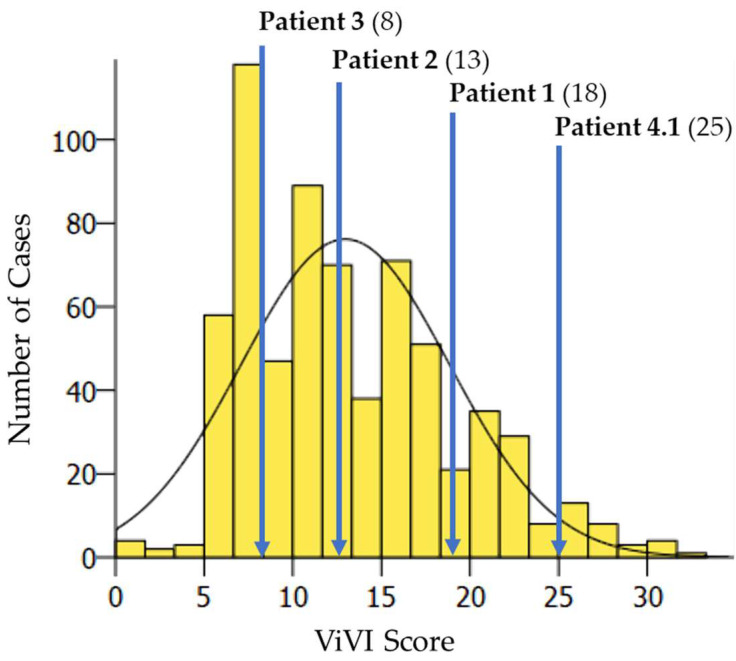
Distribution of disease severity (ViVI Scores) of cases with IV dual infection (*n* = 4) amongst all IV-positive patients (*n* = 673, median ViVI Score: 12.0, mean ViVI Score: 13.0).

Patient characteristics and clinical hallmarks of IV dual infection cases are described in Table 1. IV dual infections showed an average ViVI Score of 16 (range 8–25, ≈60th percentile of the cohort) compared to 14.5 (range 0–34), reflecting the 50th percentile of the entire cohort (*n* = 6073) [12] and 13.0 (range 0–32) reflecting the 50th percentile of all IV-positive patients for reference. The disease severity of patients with IV dual infection was within 1.75 SD of severity in the entire cohort. Figure 3 shows the distribution of disease severity (ViVI Scores) of cases with IV dual infection (*n* = 4) amongst all IV-positive patients (*n* = 673).

The mildest course of illness (maximum ViVI Score of 8) was observed in a Crohn’s disease patient under immunomodulatory therapy. All other patients with IV dual infection were previously healthy and without proof of vaccination against influenza. Only one patient was treated with a neuraminidase inhibitor.

### 3.2. Detailed Clinical Vignettes—The Dynamics of Disease Severity and Viral Load

**Figure 4:**
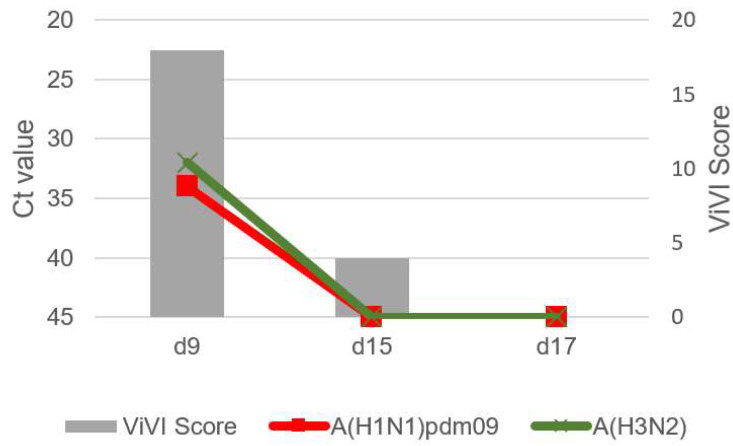
Patient 1 presents with influenza A/A virus dual infection. Virus load (left inverted vertical axis) as approximated by Ct values correlated with ViVI Scores (right vertical axis) reflecting increasing disease severity with increasing values.

**Figure 5:**
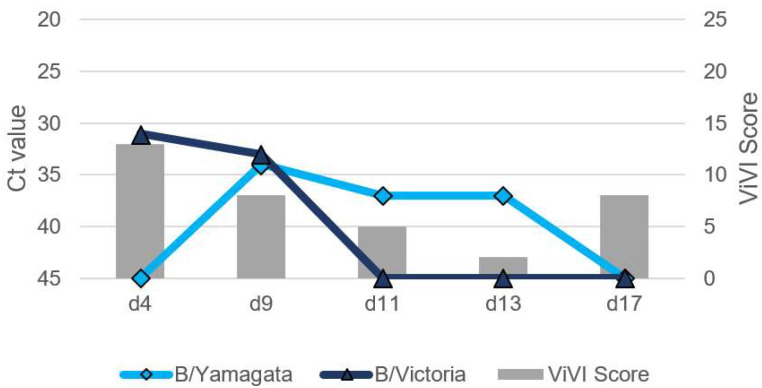
Patient 2 presenting with influenza B/B virus dual infection. Virus load (left inverted vertical axis) as approximated by Ct values correlated with ViVI Scores (right vertical axis) reflecting increasing disease severity with increasing values.

**Figure 6:**
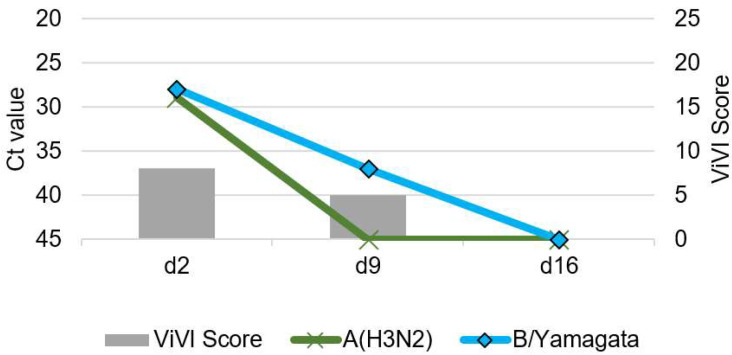
Patient 3 presenting with influenza A/B virus dual infection. Virus load (left inverted vertical axis) as approximated by Ct values correlated with ViVI Scores (right vertical axis) reflecting increasing disease severity with increasing values.

**Figure 7:**
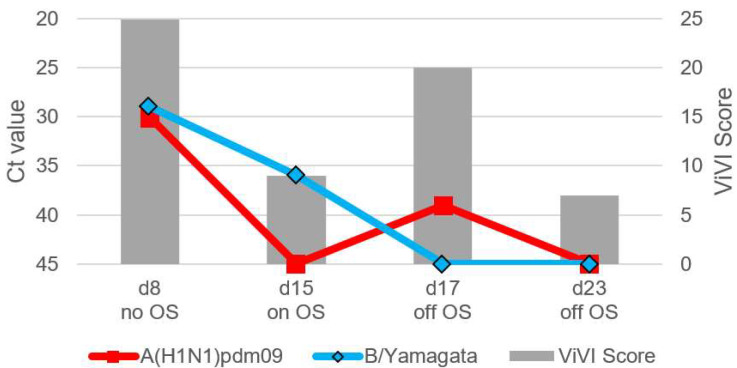
Shows Patient 4.1 testing positive for both, A(H1N1)pdm09 as well as B/Yamagata viruses at the same time. See virus load as approximated by Ct values on the left inverted vertical axis and ViVI Scores reflecting disease severity on the right vertical axis. “No OS” indicates “no treatment with oseltamivir”, “on OS” indicates “on treatment with oseltamivir”, and “off OS” “discontinuation of oseltamivir treatment”.

**Figure 8:**
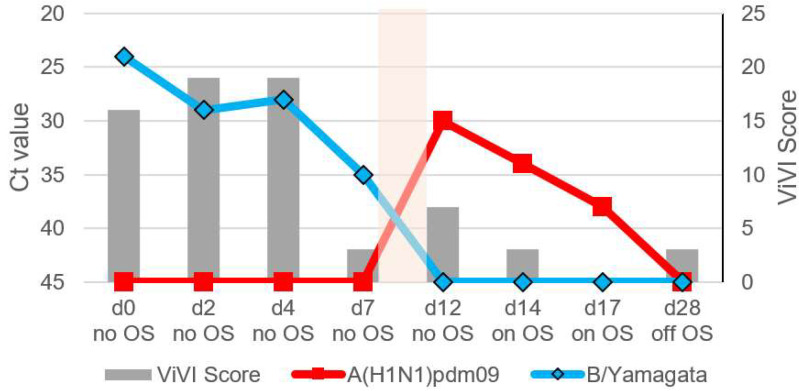
Shows Patient 4.2 initially testing positive for B/Yamagata and subsequently A(H1N1)pdm09. The shaded area in beige indicates the time frame of presumed dual infection (in between 2 visits). See virus load as approximated by Ct values on the left inverted vertical axis and ViVI Scores reflecting disease severity on the right vertical axis. “No OS” indicates “no treatment with oseltamivir”, “on OS” indicates “on treatment with oseltamivir”, and “off OS” “discontinuation of oseltamivir treatment”.

Patients with IV dual infection were studied in-depth with frequent follow-up including repeat clinical assessments, severity scoring, and viral load measurements. The dynamics of viral load (as approximated with Ct values) and disease severity (measured with the ViVI ScoreApp) are displayed in Figure 4, Figure 5, Figure 6, Figure 7 and Figure 8. The course of illness in patients with dual infection tended to be prolonged and/or complicated. ViVI Disease Severity Scores correlated well with viral loads.

### 3.3. Patient 1 (Season 2012/2013): A(H1N1)pdm09 & A(H3N2) Dual Infection

Patient 1 was a previously healthy 11-year-old female with a 5-day history of abdominal pain. Abdominal ultrasound was performed to rule out appendicitis, showing enlarged mesenteric lymph nodes. After these five prodromal days, the patient developed a fever of 101.5 °F (38.6 °C), cough, rhinitis, otitis, and difficulty concentrating (day 5). Two days later (day 7), she was hospitalized and admitted to the pediatric intermediate care unit due to an afebrile tonic seizure with prolonged loss of consciousness. On day 9, she again developed a fever, cough, and diarrhea and tested positive for influenza A(H1N1)pdm09 as well as A(H3N2) viruses. Follow-ups were performed on days 15 and 17, with otitis and rhinitis still present on day 15 and full recovery by day 17, but IV no longer be detected on either day. The highest ViVI Score was 18 on day 4 (z-score +0.58, see Figure 4). Prior to IV testing, the patient was treated with amoxicillin. No antivirals were administered. Caregivers were unable to report the vaccination status regarding influenza and no vaccination record was accessible.

### 3.4. Patient 2 (Season 2012/2013): B/Victoria & B/Yamagata Dual Infection

Patient 2 was a 5-year-old male without underlying medical conditions, presenting with a 102.4 °F (39.1 °C) fever, cough, rhinitis, bloody vomiting, and listlessness for three days. He was hospitalized and tested positive for B/Victoria on day 4 of illness with a ViVI Score of 13 (z-score -0.25). On day 9, the fever resolved, but signs of bronchopneumonia manifested. Follow-up PCR showed a dual infection with IBV of both, B/Victoria and B/Yamagata lineage (see Figure 5). No antivirals were administered. On day 17, respiratory symptoms (such as a cough) were still present, but IVs were no longer detected. The child had never been vaccinated against influenza.

### 3.5. Patient 3 (Season 2014/2015): A(H3N2) & B/Yamagata Dual Infection 

Patient 3 was a 17-year-old female with Crohn’s disease under adalimumab therapy, a disease-modifying monoclonal antibody inactivating tumor necrosis factor-alpha. She presented with cough, rhinitis, and fever up to 102.7 °F (39.3 °C) on the second day of illness. She tested positive for A(H3N2) and B/Yamagata (see Figure 6) but was in moderate condition (highest ViVI Score was 8, z-score −1.08), thus was not hospitalized. She received no antiviral treatment. Two weeks later, symptoms resolved, and IVs were no longer detected by PCR. The adolescent had never been vaccinated against influenza, although she is part of a risk group where seasonal influenza vaccine would be recommended according to the German National Immunization Technical Advisory Group (NITAG, Berlin, Germany)

### 3.6. Patient 4.1 (Season 2012/2013): A(H1N1)pdm09 & B/Yamagata Dual Infection

Patient 4.1 was a 2-year-old twin boy without known chronic illnesses. He presented with a 104 °F (40 °C) fever, cough, tachypnea, chest retractions, pulmonary rales, dehydration, and listlessness for seven days. On day 8, he was hospitalized for bronchopneumonia requiring O_2_-supplementation and tested positive for IV A(H1N1)pdm09 as well as B/Yamagata (highest ViVI Score 25 on day 8, z-score +1.75, see Figure 7). He was treated with oseltamivir from day 10-15. On day 15, fever resolved but wheezing, tachydyspnea, and weight loss persisted, and antiviral therapy was discontinued. On day 17, the patient was re-admitted to the hospital with new-onset fever at 102.2 °F (39 °C), pulmonary rales, chest retractions, dyspnea, and vomiting resulting in a 10-point increase in ViVI Scores, compared to two days prior. PCR testing on day 17 revealed a viral rebound of A(H1N1)pdm09, which had been non-detectable by PCR at the previous visit (day 15). The patient improved but required several weeks of medical rehabilitation because of weakness and persistent wheezing. The child had never been vaccinated against influenza.

### 3.7. Patient 4.2 (Season 2012/2013): B/Yamagata & Subsequent A(H1N1)pdm09 Infection

Patient 4.2 is the previously healthy two-year-old twin brother of Patient 4.1. Two days prior to his brother feeling sick, he had presented with a 104 °F (40 °C) fever, tachypnea, malaise, lethargy, difficulty feeding, and febrile seizure leading to hospitalization for two days. He tested positive for B/Yamagata. His symptoms slowly improved (highest ViVI Score was 19, z-score +0.75, see Figure 8) spontaneously, but on day 12 (=day 10 in his brother’s course of illness), he once again developed a 103.8 °F (39.9 °C) fever, pharyngitis, otitis, cough, and malaise and he tested positive for IV A(H1N1)pdm09. He was treated with oseltamivir for five days, and his symptoms resolved by day 17 with a symptomatic rebound on day 28. Due to recurrent wheezing and listlessness, the child required several weeks of medical rehabilitation. He had never been vaccinated against influenza.

## 4. Discussion

We report incidence, disease severity, and clinical follow-up of IV dual infections in a well-defined cohort of 6073 ILI patients of all cases presenting to one of Europe’s largest pediatric academic medical centers. The inception cohort approach provided a known denominator based on uniform case criteria. All patients in the QI program/cohort underwent in-depth influenza diagnostics as well as immediate standardized severity scoring at the bedside using meta-analyzable disease severity measurements and innovative mobile health technology. The result of this precision medicine approach are highly standardized real-world patient data with minimal selection and observer bias [12,23,28]. 

Prior to this investigation, our understanding of IV dual infections mainly relied on singular case reports or small retrospective case series without denominator data as would be required for the determination of incidence and risk [15,16,18,20,21,33,34]. Several retrospective studies analyzed convenience samples or sentinel surveillance sample banks with no information on timing and follow-up or whether repeated reporting from the same patient had occurred [14,17,35,36,37]. Convenience samples usually reflect patients tested in routine care [23], and national surveillance sample banks consist of a surveillance subset from a variety of sentinel sites (for example, the first three patients of the day [36]) with sparse clinical information (if any), without using consistent pre-defined case criteria or severity scoring. In a previous study, the QI program described in this paper (with a clearly defined standard operating procedure, SOP) was compared to routine care at the same hospital: The comparison showed that 61% of influenza diagnoses had, in fact, been missed in routine care, likely due to the lack of consistent clinical case criteria triggering standardized clinical assessment, data capturing, and virological testing [23].

Based on this inception cohort, we estimated an incidence rate for IV dual infection of 12/100.000 ILI patients per year with slightly increased clinical severity as reflected by an average ViVI Score ranking on the 60th percentile of the cohort (see Box 1). Comparison with other studies is limited due to the lack of prospective perennial surveillance systems over multiple years paired with standardized clinical data resulting in a standardized disease severity measure.

In the present investigation, we used the ViVI ScoreApp to measure disease severity at baseline and longitudinally. By now, the ViVI ScoreApp has been validated in nearly 10,000 patients, including in the Berlin Cohort [12], a European multi-center study [30], as well as US-based intensive care unit and community surveillance across all age groups [38]. Previously, we showed that standardized clinical severity assessments combined with microbiological and virological testing could help to enhance patient management, including stringent use of antibiotics and the development of clinical decision models [11,23,28]. With the ViVI ScoreApp, we also provide the opportunity to comprehend and track disease severity during IV dual infection, both in the same individual and between several individuals and cohorts.

Little is known about risk factors favoring the occurrence of IV dual infections. Three of four patients reported no underlying medical conditions or other risk factors for complicated ILI [3,39]. One patient was immunocompromised due to treatment for Crohn’s disease. Of note, this patient had the lowest ViVI Scores reflecting mild disease compared to the other dual infection cases. Dual infections have previously been described in transplant, cancer, and HIV patients [14,39,40,41]. However, systematic evidence is still lacking. Systematic studies in high- and low-risk populations are needed to delineate risk factors associated with severe outcomes in IV dual infection.

IV dual infections can only occur in seasons with the co-circulation of multiple IV subtypes or lineages (see Box 1). Of note, co-circulation may be a prerequisite but not a predictor of IV dual infection [42]. Even though we followed the same SOP for digital syndromic and virologic surveillance throughout six consecutive influenza seasons, 3 of 4 cases of IV dual infection were observed during the same season, i.e., 2012/2013. Of note, this was a season of high IV circulation with a great variety of different subtypes and lineages. In the 2010/2011 season, the total number of IVs detected was even higher than in 2012/2013, but the variety of subtypes and lineages was low, and we did not detect IV dual infections at that time. A recent clinical data analysis and experimental infection study found that co-circulation of specific respiratory viruses might have the potential to interfere with or even block other viral (co-) infections and IAV infection in particular [43]. The risk of IV dual infection may vary from season to season, depending on the composition of the environmental virome.

To better understand disease severity in dual or multi-viral infection, the specific timing and kinetics of infection and subsequent immune response may be relevant. In an in vitro and in vivo animal study, IAV/IDV coinfection seemed to induce time-dependent proinflammatory responses [44]. Distinct immune signatures, such as prolonged immune activation, have been linked to severe IV infection [45]. Longitudinal follow-up assessments in this digital surveillance cohort allowed us to investigate the combined kinetics of virus load (i.e., Ct values) and disease severity over time (see Box 1). In 3 out of 4 cases presented herein, IV dual infection was detected at baseline with disease severity (ViVI Scores) peaking at this exact time point. This indicates that maximum disease severity may be observed when both IV are detectable. In all three patients with IAV/IBV infection, IBV virus load declined slower compared to IAV. ViVI Scores seemed to decrease in parallel with the decline in virus load. This is in line with previous reports from our group, in which we studied the "slope of virus load decline" in IV in children in greater detail [23,24,25,27].

We showed that viral clearance happened faster in IAV than IBV infection. This study, however, described mono-infected patients under neuraminidase inhibitor therapy without viral rebound [27].

Viral rebound and slope of decline observation can only be made when virus load is studied very frequently during follow-up. This explains anecdotal reports that IAV persisted longer than IBV in one individual [19,40]. Even though virus load and disease severity seemed to correlate well in this study, prolonged shedding and disease severity cannot be attributed to specific IV species, subtype, and/or lineage. Disease severity may result from a complex interaction between virus dynamics and host-specific immune responses.

Under selective pressure, such as antiviral therapy, there might be competition among different IVs in the same individual. The present investigation provides the unusual opportunity to observe two distinct IVs responding to antiviral exposure in the same individual: The course of illness in Patient 4.1 indicates that virological rebound is not only possible but can be preceded or indicated by an unexpected rise in disease severity (as measured by ViVI ScoreApp) in a patient previously improving under neuraminidase inhibitor treatment. The constellation also suggests that repeated virology and/or resistance testing may be indicated in rebound clinical scenarios [27].

Repeated virology testing in a patient with clinical deterioration (Patient 2) also allowed us to detect B/Yamagata-B/Victoria infection, which is, to the best of our knowledge, the first published case of dual IBV infection. As a side note, the epidemiology of IBV will be of great interest during the ongoing coronavirus infectious disease 2019 (COVID-19) pandemic, with the B/Yamagata lineage appearing to be on the verge of extinction [46,47].

The occurrence of IV dual infections emphasizes the importance of multivalent vaccines to efficiently curb the influenza disease burden. Absent of a universal influenza vaccine recommendation in Germany, none of the dually infected patients in the Berlin cohort had been vaccinated against influenza. Of note, Patient 3 with underlying risk factors (immunosuppressive therapy) was unvaccinated. Universal vaccine recommendations may provide a simpler public health message helping to limit transmission and (co-)circulation of viruses. Patients and caregivers should also be informed that one IV infection does not necessarily protect against concurrent or sequential infection with other IVs, subtypes, and/or lineages.

Our study was limited to a pediatric single-center setting. Further studies are needed to elucidate the incidence of IV dual infections in the general population. The incidence of IV dual infections in this pediatric cohort was relatively low. This is a finding in and by itself highlighting the strength of systematic syndromic and virologic surveillance in capturing even rare events against a known denominator. For the small number of patients with IV dual infection, we refrained from performing statistical significance testing. Note that virological testing in the QI program was limited to two of four IV species, i.e., Influenza A virus and Influenza B virus. Therefore, we were unable to further investigate the real-world clinical impact of ICV and/or IDV (dual) infection [5,6,7]. Lastly, we might underestimate the incidence of IV dual infections for the fact that IV dual infections may occur in oligosymptomatic patients who did not seek medical care in the first place or testing IV-negative at initial examination. However, our results indicate that repeat virology testing may be useful in patients with severe, prolonged, or complicated clinical courses, as was the case in the QI program.

Future investigation linking real-world clinical data captured using innovative mobile health technology to in-depth laboratory testing will illuminate the role of host factors, including cytokine signatures during and post-infection on severe or unusual clinical presentations. This will be of particular interest in cases such as Patient 2, where ViVI Scores increased after resolution of IV shedding and in the absence of other viral and/or bacterial coinfections.

In outpatients, it may be useful to monitor the clinical course remotely. This is where patient-reported outcomes may support continuity of care. The ViVI ScoreMe App will provide this opportunity based on the epidemiological and clinical evidence learned from physician-reported severity assessments. Regular disease severity assessment may also help to elucidate in greater numbers the occurrence of (dual and mono-) infections with IV and their clinical impact in the general population, potentially strengthening IV surveillance. We are currently working with patient organizations to understand better the subjective impact of ILI symptoms in influenza, COVID, and other respiratory viruses (www.symptomsurvey.org, accessed on 3 March 2022).

We conclude that hospital-based digital syndromic and virologic surveillance deploying innovative mobile health technology at the point of care may help to unravel the real-world clinical impact of IV dual infections. Further studies are needed to elucidate the seasonality and epidemiology of IV dual infections in different geographic and demographic settings.

Box 1 **Take Home Messages.**

- IV dual infections are rare but may exist during influenza seasons with co-circulation of different subtypes and/or lineages.
- We observed complicated and/or prolonged disease with seizures, bronchopneumonia, or biphasic fever in IV dual infections; ViVI Disease Severity Scores were slightly above-average compared to the rest of the cohort.
- During follow-up, ViVI Disease Severity Scores reflected not only the clinical course of illness but also the progression of viral load (as approximated by Ct values), including during rebound.
- Combined digital (mobile health) and virological (PCR) surveillance of influenza and other respiratory viruses—ideally with longitudinal follow-up-will improve our understanding of the clinical impact of viral-viral co-infections in ILI patients.

## Figures and Tables

**Table 1:** Patient specifics of all four influenza virus dual infections. F—female, M—male, URTI—upper respiratory tract infection, LRTI—lower respiratory tract infection.

Patient	Age in Years/Sex	Influenza Virus Dual Infection	Underlying Condition	Influenza Vaccination	Hospitalization	Chief Complaint and Clinical Hallmarks	Antiviral Treatment	Baseline ViVI Score	Maximum ViVI Score
1	11/F	A(H1N1) pdm09, A(H3N2)	no	no	yes	Suspected appendicitis with mesenteric lymphadenitis, then seizure	no	18	18
2	5/M	B/Yamagata, B/Victoria	no	no	yes	Bloody vomiting, listlessness, prolonged LRTI, biphasic disease severity	no	13	13
3	17/F	A(H3N2), B/Yamagata	Crohn’s disease, immunosuppressive therapy	no	no	Prolonged URTI with fever	no	8	8
4.1	2/M	A(H1N1) pdm09, B/Yamagata	no	no	yes	Listlessness, severe/prolonged LRTI, later biphasic disease severity, wheezing	yes	25	25

## Data Availability

The data presented in this study are available on request from the corresponding author.
